# Drosophila Larval Development and Human Immunodeficiency: The Adenosine Deaminase Connection

**DOI:** 10.1371/journal.pbio.0030232

**Published:** 2005-05-24

For most healthy individuals, infection triggers a rapid immune response that repels the invaders. But for those rare individuals born without the immune system cells (lymphocytes) that recognize and kill pathogens, bacterial, viral, or fungal encounters can result in recurrent infections that are more life-threatening and less responsive to treatment than similar infections in normal infants. In the past, all that could be done for children with severe combined immunodeficiency (SCID) was to protect them from infections by cocooning them in sterile plastic bubbles, which gave the disease its common name: bubble-boy syndrome. Nowadays, the treatment of choice, provided a suitable donor is available, is bone-marrow or stem-cell transplantation, which provides SCID children with a functioning immune system.

Mutations in at least nine genes can cause human SCID, but 20% of cases are caused by a deficiency of the enzyme adenosine deaminase. This enzyme, which is present in all organisms, converts adenosine and deoxyadenosine to inosine and deoxyinosine, respectively. When adenosine deaminase is missing, its substrates (adenosine and deoxyadenosine) accumulate, and this is thought to cause the complete breakdown in immune defense characteristic of SCID.

To explore the role of adenosine deaminase in a tractable model system, Peter Bryant and his colleagues have now developed a Drosophila model by disabling the expression of a protein—called adenosine deaminase-related growth factor A (ADGF-A)—that serves as a major adenosine deaminase in the fly. In flies lacking ADGF-A enzymatic activity, adenosine and deoxyadenosine concentrations increase in the larval hemolymph, the circulatory fluid or “blood” of insects. Lack of the enzyme, the researchers report, caused larval death associated with the disintegration of the fat body (the adipose tissue spread throughout the body of the insect), melanotic tumors, and delays and defects in development.

The first two effects, fat body disintegration and the presence of melanotic tumors, are directly attributable to dysregulation of hemocytes (fly blood cells) in the mutant animals. It turns out that in *adgf-a*-mutant larvae, hemocytes are released prematurely from the lymph glands (the organs where hemocytes are produced and stored). These prematurely released hemocytes then cause fat body disintegration and formation of melanotic tumors; however, if ADGF-A expression is selectively restored in the lymph glands, then hemocytes are not prematurely released, and the larvae survive and develop without the tumors or fat body disintegration.

Bryant and his colleagues reasoned that the elevated adenosine might have direct effects on fly development aside from the dysregulation of hemocytes, so they examined the development of *adgf-a* mutant flies that also lacked a functional adenosine receptor (*adoR* mutants). They found that *adgf-a/adoR* mutant larvae were able to survive and continue development to adulthood, although these animals still experienced fat body disintegration and some melanotic tumors. These results suggest that the second consequence of ADGF-A deficiency, delayed development, is caused by the elevated adenosine in the animals signaling through adenosine receptors. [Fig pbio-0030232-g001]

**Figure pbio-0030232-g001:**
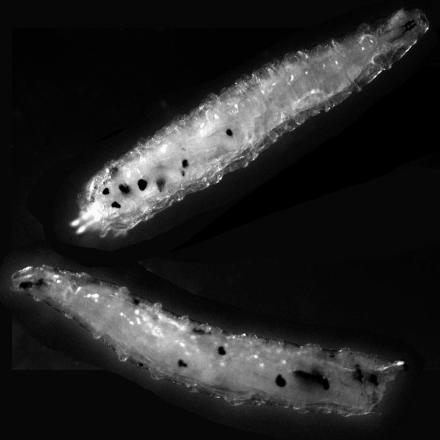
Drosophila *adgf-a* mutant larvae with melanotic tumors in their body cavities

Altogether, these results establish *adgf-a* flies as a useful model system for unraveling the many effects that adenosine and deoxyadenosine have on cellular physiology in general and on the immune system in particular. Because hemocyte release from lymph glands and delays in development also occur in response to infection, the authors hypothesize that adenosine might be involved in controlling hemocyte release and postponing development when fly larvae are challenged by microbial attacks. Future experiments in this model system should provide important clues to the pathology of adenosine deaminase deficiency–associated SCID and should also advance our understanding of how adenosine acts as a stress hormone during infections in individuals with normal immune systems.

